# Effect of Zinc on the Oxidative Stress Biomarkers in the Brain of Nickel-Treated Mice

**DOI:** 10.1155/2019/8549727

**Published:** 2019-09-02

**Authors:** Jurgita Šulinskienė, Rasa Bernotienė, Dalė Baranauskienė, Rima Naginienė, Inga Stanevičienė, Artūras Kašauskas, Leonid Ivanov

**Affiliations:** ^1^Neuroscience Institute, Lithuanian University of Health Sciences, Kaunas LT-50161, Lithuania; ^2^Faculty of Medicine, Medical Academy, Lithuanian University of Health Sciences, Kaunas LT-50161, Lithuania

## Abstract

The overexposure to nickel due to the extensive use of it in modern technology remains a major public health concern. The mechanisms of pathological effects of this metal remain elusive. The present study was devoted to evaluate the effect of nickel on the oxidative state of the brain cells of mice and to assess whether zinc as redox state modulator could efficiently protect cells against nickel's neurotoxicity. As oxidative stress biomarkers in the present study, we have measured the concentrations of reduced glutathione, metallothioneins, and malondialdehyde and the activity of the enzyme *δ*-aminolevulinate dehydratase. For the single metal exposure, mice were i.p. injected once with solutions of NiCl_2_ and/or ZnSO_4_; repeated exposure was performed i.p. injecting metal salt solutions for 14 days (once a day). The control mice received i.p. injections of saline. Results of our study demonstrate that single and 14 days of Ni^2+^ exposure decreased reduced glutathione and increased malondialdehyde contents in the brain of mice. Repeated Ni^2+^ administration significantly inhibited *δ*-aminolevulinate dehydratase while increasing brain metallothionein concentration at both exposure periods. Zinc exhibited a protective effect against nickel-induced glutathione and lipid peroxidation in brain cells of mice at both intervals of time, while repeated exposure to this metal significantly raised the brain metallothionein content. Repeated Zn^2+^ pretreatment protected *δ*-aminolevulinate dehydratase from Ni^2+^-induced inhibition and significantly increased metallothionein concentration at both investigated time intervals.

## 1. Introduction

Nickel is a transition metal found in the Earth's crust in combination most usually with iron, sulphur, oxygen, or arsenic. Ni compounds and metallic form of this metal are used in a wide variety of industrial and commercial applications [1, 2]. In combination with some other metals, Ni is used to form alloys, to produce coins, ceramics, steel, jewellery, battery, medical devices, electroplating, orthodontic appliances, and many others [3, 4]. Extensive use and high consumption of Ni-containing products inevitably lead to a high level of contamination and the environmental pollution by Ni and its derivatives [5]. Pollution increases human exposure to Ni primarily via inhalation and ingestion; however, wearing the jewellery may also result in cutaneous absorption of Ni [6].

Ni has been added to the list of essential trace elements quite recently; however, by now, there exists a substantial list of Ni-required enzymes [2, 7, 8]. It was considered as an essential element based on reports of Ni necessity for plants and deficiency in some animal species; however, the functional importance of Ni and its physiological relevance in humans yet remain unclear, and deficiency was never reported either [2, 3, 9]. Although healthy human body contains up to 10 mg of Ni and some data suggest that it might be involved primarily in the regulation of liver function, related to normal growth, Fe homeostasis, and red blood cell production, the exact role of Ni is still unclear [10, 11].

Toxicity of Ni depends on the route of the exposure as well as solubility of Ni compound and has a number of possible mechanisms, including disruption of trace elements and iron homeostasis, interaction with macromolecules, disturbance of development and energy metabolism, and induction of oxidative stress [6, 11, 12]. Since inhaled Ni is mainly accumulated in the cerebral cortex and whole brain, the nervous system is widely considered as a major target of Ni toxicity [6, 7]. Some studies with animals showed neurobehavioral changes, degeneration of neurons in the hippocampus and cerebral histopathological changes, and alteration of cognitive and locomotor behaviors in rats; however, current knowledge of Ni's neurotoxicity mechanisms still remain limited [3, 7].

Zinc is the second most abundant biometal after iron; it is the most commonly utilized metal cofactor, required for about 16% of all enzymes [13, 14]. Nearly 10 percent of the proteins, encoded in human genome require Zn for their proper structure and function [14]. Zn acts as antioxidant, which protects from the oxidation sulfhydryl groups of enzymes and other proteins, thus stabilizing these biomolecules [15]. The precise mechanism of antioxidant action of Zn is not completely understood; however, there are suggestions that being redox stable, Zn replaces redox active metals at critical cellular or extracellular sites and/or induces synthesis of cysteine-rich protein metallothioneins [15, 16].

Reduced glutathione (GSH) is the most abundant water-soluble tripeptide within the cell. The thiol group from cysteine provides GSH great reducing power, while high intracellular concentration makes it one of the major components of the cellular antioxidant system [17]. GSH deficiency is considered to be one of the earliest biochemical indicators of neuronal oxidative damage, degeneration in aging or certain mental disorders [17, 18].

*δ*-Aminolevulinate dehydratase (*δ*-ALAD) is a Zn-dependent metalloenzyme, rich in thiol groups, and therefore, sensitive to all chemical agents that are inclined to interact with them. Proximity between thiol groups renders *δ*-ALAD extremely sensitive to inhibition by heavy metals that displace Zn and/or oxidize the sulfhydryl groups [19]. Recent studies propose this enzyme as one of the most sensitive to cellular levels of Zn and a marker protein of oxidative stress [19, 20].

Metallothioneins (MTs) are cysteine-rich low molecular weight, metal-binding proteins, which are involved in many physiological and pathophysiological processes [21, 22]. They have been proposed to protect cells against metal toxicity, regulate homeostasis of trace elements, and provide a shield against reactive oxygen species (ROS) and are considered as one of the most important markers to monitor environmental metal contamination [21–23].

The excess ROS exposure is known to cause oxidative damage to cellular components involving polyunsaturated fatty acid residues of phospholipids, which are extremely sensitive to oxidation [24, 25]. As malondialdehyde (MDA) is a major endogenous product of lipid peroxidation (LPO), its content in membranes is one of the most usable indicators of this process [26, 27].

Our previous studies performed with mice liver and blood, as well as few studies accomplished by other scientists, proposed the existence of some kind of competition between Ni^2+^ and Zn^2+^ [15, 28–30] as well as a possible protective role of Zn against oxidative stress. However, detailed studies of the mechanisms of the interaction of these metals in the brain are almost nonexistent while research on the ability of Zn to protect the brain from Ni is very scarce.

Thus, the aim of this study was to investigate the effect of Ni^2+^ on oxidative stress markers of the brain cells of mice and to evaluate whether Zn^2+^ as redox state modulator could efficiently protect cells against Ni's neurotoxicity. Therefore, the responses of four biomarkers, i.e., contents of GSH, MDA, and MT and activity of *δ*-ALAD, were examined.

## 2. Materials and Methods

4–6-week-old out-bred white laboratory mice, each weighing from 20 to 25 g were used in these experiments. All experiments were performed according to the Republic of Lithuania Law on the Care, Keeping and Use of Animals (License of State Veterinary Service for Working with Laboratory Animals No. 0200). Mice were randomly assigned into 3 metal exposure groups plus a control group which received i.p. injections of saline. Mice of Ni and Zn exposure groups received an i.p injections of corresponding amounts of NiCl_2_ and ZnSO_4_ dissolved in saline as shown in Table 1. Mice of the Zn+Ni exposure group were i.p. injected with ZnSO_4_ and after 20 min with NiCl_2_ solutions in corresponding doses (see Table 1). There were two models chosen with a different duration of mouse exposure to the metals. For the acute single metal exposure, the exposure time was set at 24 hours and mice were injected once. For the acute repeated exposure, mice were i.p. injected for 14 days (once a day) with metal salt solutions (see Table 1).

All the animals of each group were anesthetized and terminated 24 h after the last dosing, according to the rules defined by the European Convention for the Protection of Vertebrate Animals Used for Experimental and Other Purposes. The brain was removed, washed, weighed, and cooled on ice.

Brain tissues were homogenized with 6 volumes *w*/*v* of 5% trichloroacetic acid. The homogenate was centrifuged at 10,000×g for 7 min.; obtained supernatant was further used to asses GSH concentration. The content of GSH of a brain tissue was measured by reaction with 5,5′-Dithiobis-(2-Nitrobenzoic Acid) (DTNB), known as Ellman's Reagent, to give a yellow colored compound that absorbs at a wavelength of 412 nm. [15]. Every single sample contained 0.6 mM DTNB in 0.2 M sodium phosphate (pH 8.0), supernatant fraction, and 0.2 M phosphate buffer [15]. GSH content was expressed as *μ*mol/g of the wet weight of the tissue.

The activity of *δ*-ALAD was examined by a method of Berlin and Schaller modified by Sassa [31, 32]. Brain tissues were homogenized with 7 volumes *w*/*v* 0.15 M NaCl (pH 7.4). The brain homogenate was centrifuged at 15,000×g for 15 min.; obtained supernatant was further used to asses *δ*-ALAD activity. For the determination of reaction product porphobilinogen, Ehrlich's reagent was used [31, 32]. Absorbance was measured at the wavelength of 555 nm. Activity of *δ*-ALAD was expressed in nmol/h/mg of protein.

Metallothioneins were assayed in mouse brain according to the method proposed by the United Nations Environment Program [33]. Tissues were homogenized with 3 volumes *w*/*v* sucrose-TRIS buffer (pH 8.6), resulting homogenate was centrifuged at 30,000×g for 20 min. High molecular weight proteins precipitated using cold (-20° C) absolute ethanol and chloroform and centrifuged again at 6,000×g for 10 min. The obtained supernatant (after elimination of low molecular weight soluble thiols) was further used to determine MT concentration by evaluating the –SH residue content by a spectrophotometric method, using Ellman's reagent. The absorbance of the supernatant was evaluated at wavelength 412 nm. GSH was used as a standard for quantification of MT in the sample, taking into account that GSH contains one cysteine per molecule [33].

To determine the extent of LPO, the content of MDA was measured. MDA is known to form adduct with 2 thiobarbituric acid (TBA) molecules to produce a pink colored pigment [34]. Tissues of a brain were homogenized with 9 volumes *w*/*v* of cold 1.15% KCl. Resulting homogenate was added to 1% H_3_PO_4_ and 0.6% TBA aqueous solution. The reaction mixture was heated for 45 min in a boiling water bath, then cooled, added n-butanol, and mixed thoroughly. The butanol phase was used to determine light absorbance at 535 and 520 nm. [34]. The results are expressed as nmol/g of the wet weight of the tissue.

Statistical analysis was performed using a statistical software package (Statistica 6.0). Statistical analysis of the obtained data is expressed as the mean (M) ± standard error of mean (SE). To determine the existence of statistically significant differences between the means of groups, Student's *t*-test was performed. *p* value less than 0.05 was considered statistically significant.

## 3. Results

**Figure 1:**
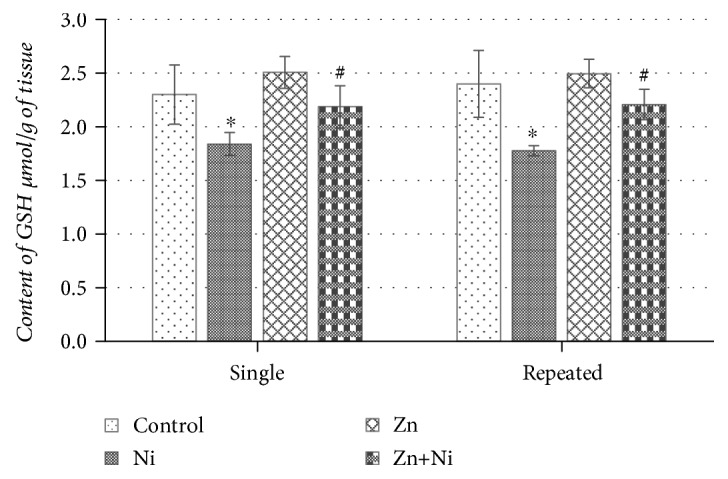
Content of GSH in the brain of mice after single and repeated (14 days) Ni^2+^ and/or Zn^2+^ exposure. Data represents results of 8–12 separate experiments. ^∗^*p* < 0.05 vs. the control group of mice; ^#^*p* < 0.05 vs. the group of Ni^2+^-treated mice.

According to the results, represented in Figure 1, a single dose of NiCl_2_ caused a statistically significant decrease of GSH content in the mouse brain as compared to the control. Mouse treatment with ZnSO_4_ solution did not have any effect on the content of GSH; however, pretreatment with ZnSO_4_ 20 min before NiCl_2_ injections attenuated the effects of Ni^2+^ and returned GSH content in the cells of the brain to the control level (Figure 1). Continuous 14-day exposure to Ni^2+^ reduced brain GSH concentration even further than the single NiCl_2_ administration and as compared to the control group of mice (*p* < 0.05) (Figure 1). Repeated ZnSO_4_ administration did not provide any effect on the brain GSH level; however, repeated pretreatment with Zn^2+^ before NiCl_2_ injections seemed to have an appreciable protective effect against Ni^2+^-induced GSH oxidation, returning its content to the level of the control mouse group (Figure 1).

**Figure 2:**
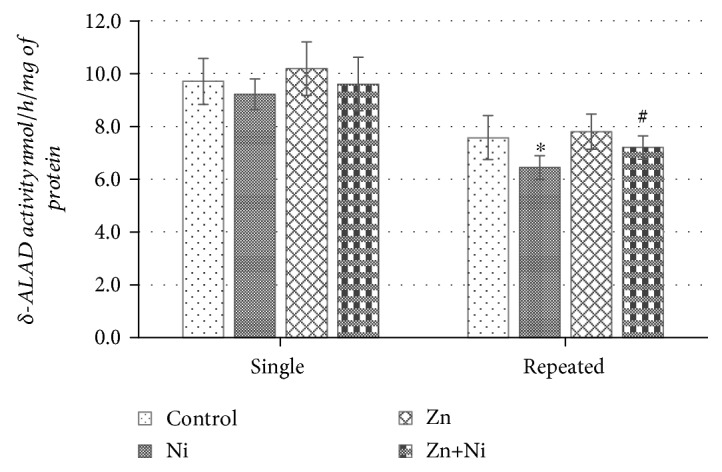
*δ*-ALAD activity in the brains of mice after single and repeated (14 days) Ni^2+^ and/or Zn^2+^ exposure. Data represents results of 8–12 separate experiments. ^∗^*p* < 0.05 vs. the control group of mice; ^#^*p* < 0.05 vs. the group of Ni^2+^-treated mice.

According to the data presented in Figure 2, neither single NiCl_2_ nor ZnSO_4_ or both metal coadministration had an appreciable impact on *δ*-ALAD activity in the brain of mice. Repeated administration of Ni salt (Figure 2) seemed to significantly suppress brain enzyme activity as compared to the control group of mice. Repeated ZnSO_4_ administration, just as the single one, had no tangible effect on the activity of *δ*-ALAD; meanwhile, repeated mouse pretreatment with Zn^2+^ before NiCl_2_ injections seemed to attenuate a suppressing effect of Ni^2+^ and returned brain enzyme activity back to the control level (Figure 2).

**Figure 3:**
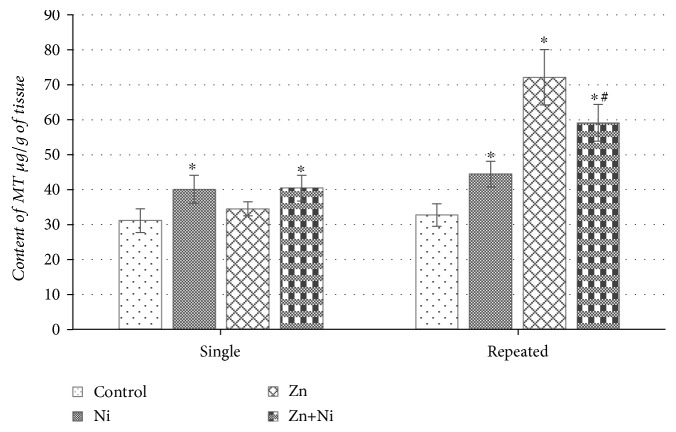
Content of MT in the brain of mice after single and repeated (14 days) Ni^2+^ and/or Zn^2+^ exposure. Data represents results of 8–12 separate experiments. ^∗^*p* < 0.05 vs. the control group of mice; ^#^*p* < 0.05 vs. the group of Ni^2+^-treated mice.

The data of Figure 3 shows that brain MT content significantly increased in animals once treated with Ni^2+^ however remained at the control level after single ZnSO_4_ administration. In the brain of animals once administered by both metals, the content of MT was significantly higher as compared to the control group of mice (*p* < 0.05). Further results (Figure 3) shows that 14 days of NiCl_2_ treatment, even more than a single Ni^2+^ administration increased the metallothionein content in the brain of mice (*p* < 0.05). Though a single dose of Zn^2+^ did not seem to have any significant effect on MT quantity, repeated exposure to this metal apparently increased the brain MT content in comparison to control (*p* < 0.05). The same tendency was observed in the ZnSO_4_ pretreated animal group; repeated exposure to both metals significantly increased MT concentration as well (Figure 3).

**Figure 4:**
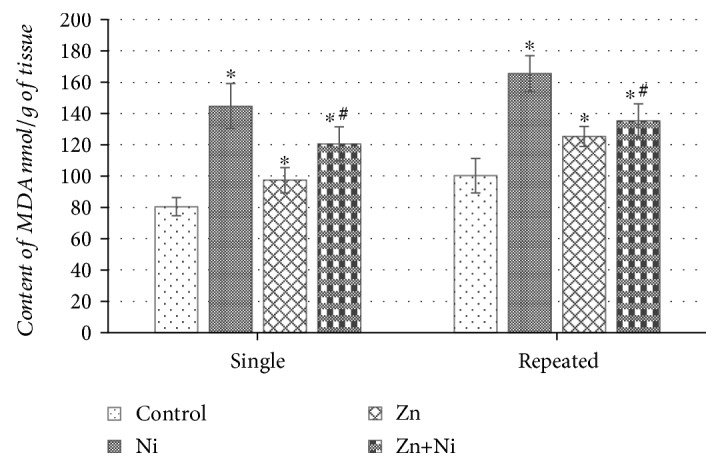
Content of MDA in the brain of mice after single and repeated (14 days) Ni^2+^ and/or Zn^2+^ exposure. Data represents results of 8–12 separate experiments. ^∗^*p* < 0.05 vs. the control group of mice; ^#^*p* < 0.05 vs. the group of Ni^2+^-treated mice.

Results represented in Figure 4 indicate that a single dose of Ni^2+^ significantly increased the MDA content in the brain of mice, as compared to the control (*p* < 0.05). Although single ZnSO_4_ administration seemed to induce some LPO increasing brain MDA content (*p* < 0.05), single Zn^2+^ pretreatment before NiCl_2_ injection subdued LPO (*p* < 0.05) comparing with the Ni^2+^ group, though could not restore it to the control level (Figure 4). Repeated injections of NiCl_2_ just like a single, this metal exposure increased MDA amount in the brain in comparison to control (*p* < 0.05) (Figure 4). Continuous administration of ZnSO_4_ had a very similar effect to that of a single dose; Zn^2+^ tended to slightly activate LPO (*p* < 0.05). However, repeated Zn^2+^ pretreatment before NiCl_2_ injections partially protected against Ni^2+^ induced brain lipid oxidation reducing the MDA content as compared to the nickel-affected group of mice (*p* < 0.05).

## 4. Discussion

Our attention in Zn/Ni interaction was drawn due to the fact that environmental Ni contamination is a widespread phenomenon in recent times. The possible impact and effects of Ni on physiologically essential metal-dependent biological systems and its metabolic pathways in the brain are not yet fully understood.

In order to assess the antioxidant status of the brain, we have evaluated the alterations of GSH, MDA, and MT contents as well as the activity of ALAD as reliable oxidative stress biomarkers, since these parameters are closely related and interdependent [26, 35–38].

There are several reasons that makes the brain especially prone to oxidative stress. Although the mass of the brain is relatively small compared to the rest of the body, it consumes up to one-fifth of the total oxygen consumed by the body [39]. Although the O_2_ is absolutely essential for the brain, it also is considered as potentially toxic mutagenic gas, since it is able to give rise to free radical and nonradical species, such as superoxide anion (O_2_^·-^), hydrogen peroxide (H_2_O_2_), and hydroxyl radical [38, 39]. In the healthy brain, where the redox balance is maintained, the resulting ROS are beneficial and even necessary; however, redox balance disturbance causes a condition known as oxidative stress, which is closely related to neurodegeneration [39, 40]. High O_2_ exposure, considerable levels of polyunsaturated n-3 fatty acids, generous Fe and Cu stores, and a quite weak antioxidant system with low levels of the antioxidant enzymes make the brain very susceptible to oxidative stress [39, 40]. It has been shown that heavy metals penetrate across the blood brain barrier causing oxidative stress and alterations in the metabolism of some proteins involved in neurodegeneration [22].

It has been suggested that transition metal Ni disturbs the homeostasis of Fe, leading to iron accumulation, which activates free radical generation through Haber-Weiss and Fenton chemistry [5, 41]. By substituting for Fe, Ni supposedly inhibits many Fe-containing enzymes; it has been shown to suppress the activities of several tricarboxylic acid cycle and electron transfer chain oxidoreductases, thus generating free radicals due to disrupted mitochondrial respiration [6, 28]. Data suggest possible Ni's inhibition of Zn metalloenzymes—several Zn-metallopeptidases had shown susceptibility to micromolar amounts of Ni [28]. By linking with cysteine, histidine, or glutamate residues, Ni seems to have the ability to inhibit a wide range of enzymes that do not require a metal for their catalytic activity [28]. Despite ROS formation, disturbed electron transfer in mitochondria is followed by inhibition of oxidative phosphorylation, thus forcing cells to turn anaerobic glycolysis on. Restricted production of ATP in nerve cells is known to cause brain tissue dysfunction and neurological disorders [5, 6]. Studies suggest that beside some respiratory chain enzyme inhibition, Ni is able to suppress the gene expression of them thereby exacerbating the damage to the mitochondria even further [41]. Since mitochondria are the prime target for Ni exposure, oxidative damage done to it is considered as one of the key causes of Ni toxicity [3, 6].

Glutathione is a water-soluble tripeptide of vital importance in millimolar concentrations found in cells of various tissues and body fluids [42]. It is the most abundant intracellular antioxidant molecule, containing a sulfhydryl group which is critical for the biological activity of GSH [43]. GSH has been suggested as the primary defence against metal cation toxicity, while it has many different functions including heavy metal and xenobiotic detoxification, reduction of sulfhydryl groups of thiols, neutralization of ROS, and regeneration of other essential antioxidants [44]. Depending on the redox status of the cell, the tripeptide exists in a reduced or oxidized form (GSSG). The relative amounts of each form (ratio of GSH/GSSG) reflect the cellular oxidative index and serve as measurable biomarkers of redox status of the cell or whole body [42, 43].

The intracellular level of GSH is an important factor in the process of cellular resistance to Ni^2+^ [44]. Our previous study demonstrated a statistically significant decrease (by 20%) of GSH content in the liver as well as the blood of mice after 24 hours of NiCl_2_ exposure. Recent studies accomplished by other scientists report a considerable GSH depletion even after single Ni^2+^ exposure [15].

It was observed that electroplate workers with exposure to high levels of Ni^2+^ had significantly lower levels of GSH than the others [27]. Consequently, a significant brain GSH content decrease after single as well as repeated exposure to Ni observed in our present study is likely to evidence the oxidative damage to the brain induced by this metal. Thus, the results of our study are corroborating the conclusions of scientists who claim intracellular GSH depletion as the result of Ni-induced toxicity [5]. Some researchers, however, have showed that Ni does not diminish but even increases GSH levels in the cell. Topal et al. found that 21-day oral NiCl_2_ treatment significantly increased GSH levels in brain tissues of rainbow trout [6, 7]. This GSH content increase could be the outcome of the adaptive mechanism, when synthesis of this tripeptide-antioxidant increases as a response to cell oxidative damage caused by toxicity of metals [7]. To the control level, restored concentration of GSH in the brain of the Zn-pretreated mouse group, observed in our present study, might be the outcome of both—Zn's ability to compete with Ni or its importance in the regulation of GSH synthesis, due to the increased expression of glutamate-cysteine ligase which is the rate-limiting enzyme of glutathione de novo synthesis [36, 37, 45].

Since thiol groups have been proposed to be the first and primary targets of heavy metal exposure and it has also been shown that some of the zinc-dependent enzymes are vulnerable to micromolar amounts of Ni, next object of our study was investigation of the possible damage caused by Ni to Zn-dependent enzymes such as *δ*-ALAD [35, 40].

*δ*-ALAD is a metalloenzyme with 3 adjacent sulfhydryl groups in its active site that requires Zn for its activity, therefore might be sensitive to the substances that compete with Zn or to those that oxidize -SH groups [20]. It is a widely distributed enzyme in nature that plays a primary role in most aerobic organisms since it catalyses condensation of two molecules of *δ*-aminolevulinic acid (*δ*-ALA) to form porphobilinogen in the heme biosynthesis pathway [26]. Recent studies have indicated that due to its high sensitivity to prooxidant situations, *δ*-ALAD can be a reliable universal biomarker of oxidative stress—various observations evidence a negative correlation between enzyme activity and level of oxidative stress [20, 26]. *δ*-ALAD inhibition due to thiol group oxidation leads to *δ*-ALA autoxidation, further *δ*-ALAD inhibition, additional O_2_^·-^ generation, and antioxidant system depletion [26, 46]. Excess *δ*-ALA in the brain disrupts *γ*-aminobutyric acid/glutamate system causing neurotoxicity and cell death [19, 46] while decreased heme biosynthesis is known to cause neuronal cell dysfunction, since heme is critical for neuronal cell growth, differentiation, and survival [47]. Literature data show that Ni is especially prone to bind to amino acids like cysteine and histidine, small peptides like glutathione, and amine-containing compounds thus accelerating the rate of oxidative damage [28]. In our previous research [30], we ascertained that single exposure to NiCl_2_ suppressed the activity of *δ*-ALAD in the liver and the blood of mice, while Zn-pretreatment diminished this effect of Ni. Repeated NiCl_2_ administration decreased *δ*-ALAD's activity in the liver of mice, though Zn did not protect enzyme from Ni-induced inhibition [30]. Other studies suggest that some heavy and transition metals cause inhibition of the *δ*-ALAD activity [19, 20] that supports the inhibitory effect of Ni on the enzyme activity after 14 days of NiCl_2_ treatment observed in our study. The fact that the single NiCl_2_ exposure did not induce any appreciable effect on *δ*-ALAD activity might be associated with the decreased levels of GSH found in the brain of mice after single Ni^2+^ treatment or with compensatory mechanisms of other antioxidant system components such as ascorbic acid or antioxidant enzymes [27, 48]. Literature suggests that due to antioxidant capacity, GSH is able to attenuate free radical generation as well as LPO, reduce oxidant burden, and partially restore enzymatic *δ*-ALAD activity, suppressed by different prooxidants [19, 20, 26].

The structure of *δ*-ALAD is characterised by vicinal cysteine residues in its active site, which are involved in the coordination of cofactor Zn^2+^ [20]. Literature suggests that close spatial arrangement of thiol groups determines the specific sensitivity of the enzyme to oxidation. Since Zn is involved in sulfhydryl group stabilization, its removal or replacement is known to accelerate enzyme autooxidation [19]. The protective effect of Zn^2+^ on the activity of the enzyme after fourteen days of metal exposure determined in our study confirms the findings of other scientists who claim Zn's ability to protect *δ*-ALAD from the inhibition, which indicates that enzyme activity suppression occurs not only by thiol group oxidation but also by Zn displacement [20].

Well-known antioxidants and heavy metal contamination biomarkers, cysteine-rich protein MTs, are also involved in maintaining some biologically essential metal ion homeostasis [22, 49]. Scientific data indicate that MTs act as antioxidant by scavenging ROS; they are capable of reducing oxidative tissue damage and helping the cells to withstand heavy metal toxicity [50, 51]. There was an established direct correlation between intracellular heavy metal and MT concentration in organs such as the liver, kidneys, and gills of aquatic animals [22, 49]. Other scientific sources show the ability of Ni to induce MTs' synthesis in various organisms and in cultured cells [52]. However, there is a sparse data on the ability of MT to reduce heavy metal-induced oxidative nerve tissue damage.

Although MTs have a particular affinity for Zn^2+^, in excess of some transition or heavy metals, Zn^2+^ can be easily displaced by them. Up to eighteen different metals are known to have the ability to associate with MTs [51]. Zn^2+^ from the Zn-MT complex can be ejected not only by certain metals; it is known by now that Zn might be displaced from MT's as a response to the exposure to ROS. Literature indicates that MT's ability to couple or release Zn^2+^ depends very much on the redox state of the cell [23]. The free Zn^2+^ merges with the six Zn fingers of metal response element-binding transcription factor-1 (MTF-1), thus activating it and inducing the expression of MT genes, thereby increasing their concentration in the cell [51]. That could explain the same trends in the increase of MT concentrations observed in our study after the repeated Zn^2+^ treatment as well as after acute and prolonged exposure to both metals on the brain of mice. It is interesting to note that an apparent increase in the content of MT determined in the brains of Zn+Ni-treated mouse group correlates with the restored content of GSH and regained enzyme *δ*-ALAD activity, which supports the idea of Zn's and MT's ability to suppress toxicity of Ni^2+^.

Since Zn excess, just as the deficiency is damaging to the cells—the cellular Zn^2+^ concentration must be precisely regulated. Scientific data affirm that this balance is supported mainly by MT and MTF-1 and might be disturbed as a response to the stress of the cell, caused by different stressors, like prooxidants and heavy metal ions [51]. It has been shown that MTF-1 regulates the MT gene expression as a response not only to changes in Zn but also to changes in other heavy metal, such as Ni concentrations [2, 51]. It was also demonstrated that exposure to NiSO_4_ activates protein phosphatase 2A, which induces MTF-1 dephosphorylation that is required for transcription factor translocation to nucleus to induce the expression of MT [2]. Increase in brain MT content, obtained in our study after single and repeated Ni^2+^ exposure, seems to be consistent with other researchers' data confirming Ni's ability to induce MT synthesis not only in the liver or kidneys but obviously in the brain as well.

The brains are enriched by redox active transition metals, like Fe^2+^ and Cu^2+^, consume lots of O_2_, and basically are composed of easily oxidizable polyunsaturated fatty acids. Neurons possess around 50 percent lower cytosolic GSH levels; as compared to cells of other tissue, enzymatic GSH-dependent antioxidant system in the brain is modest which determines nerve tissue vulnerability to oxidative stress-induced LPO [39]. Although Ni, compared with other metals such as Fe, is not very effective in Fenton chemistry, its reactivity seems to be enhanced when it is chelated by oligopeptides or histidine [12]. Ni is known to strongly interfere with Fe homeostasis, leading to Fe^2+^ accumulation which in turn induces oxidative stress through Fenton and Haber-Weiss reactions and initiates LPO [39, 48]. Topal et al. showed that after three weeks of daily treatment, Ni in a dose-dependent manner significantly increased rainbow trout brain LPO, which caused demyelination and necrotic changes in some brain areas [7]. Other researchers notice that beside LPO, Ni depletes the intracellular antioxidants and significantly increases the activity of antioxidant enzymes like glutathione reductase and catalase [3, 12]. Lipid peroxides formed due to LPO are converted to their corresponding alcohols by the glutathione peroxidases, which convert GSH into oxidized glutathione disulphide [27]. Our observed overlap of MDA content elevation with expend of GSH, after single and continuous Ni^2+^ exposure, appears consistent with other researchers' findings confirming Ni's ability to induce oxidative damage, which significantly promote LPO and antioxidant system depletion not only in other organs but also in the brain [6, 7, 12].

According the data of our experiments, Zn^2+^ pretreatment before Ni^2+^ injections seemed to significantly reduce brain LPO as compared to the NiCl_2_-treated group of mice. A decrease in MDA might involve Zn's ability to induce synthesis of glutathione, which is a coenzyme of glutathione peroxidase, thus increasing antioxidant enzyme activity [36]. Zn's ability to compete with Fe and Cu for the binding sites in the cell membranes could be another explanation of brain lipid protection, since both Fe and Cu are able to induce the formation of free radicals from lipid peroxides. In this case, the replacement of Fe or Cu with redox stable Zn could prevent cell from free radical formation [36]. Zn acts a cofactor for another antioxidant metalloenzyme superoxide dismutase (SOD) which promotes two O_2_^·-^ radical conversion to H_2_O_2_ and O_2_, thus reducing toxicity of ROS [36]. An increase in SOD activity might appear as a result of Ni^2+^/Zn^2+^ competition for enzyme binding or to Zn's ability to activate SOD synthesis at a transcriptional level [28, 53]. A number of pathologies like neurodegeneration and myocardial injury were observed in SOD-deficient mice, since the enzyme is indispensable in protection against oxidative damage [48].

## 5. Conclusions

Single and repeated exposure to Ni^2+^ resulted in an expended resource of GSH as well as enhanced LPO in mouse brain. Single i.p. injection of NiCl_2_ did not affect enzyme *δ*-ALAD activity; however, single as well as repeated Ni^2+^ administration significantly inhibited enzyme and increased the content of MT. Zn^2+^ provided the protective effect against Ni^2+^-induced GSH depletion and LPO at both exposure periods. After continuous pretreatment, Zn^2+^ managed to return Ni-suppressed *δ*-ALAD activity back to the level of control; MT content after prolonged both metal administration remained increased.

In summary, Zn definitely has shown some protective mechanisms against toxicity of Ni; however, further studies on the Ni-Zn interaction, including the response of brain antioxidant enzymes, would potentially help to further understand the effects of these metals on the redox state of the brain.

## Figures and Tables

**Table 1:** Metal exposure groups and doses of metal solutions.

	Ni	Zn	Zn+Ni
Acute single metal exposure	96 *μ*mol Ni^2+^/1 kg b.w.	24 *μ*mol Zn^2+^/1 kg b.w.	24 *μ*mol Zn^2+^/1 kg b.w. and 96 *μ*mol Ni^2+^/1 kg b.w.
Acute repeated metal exposure	19 *μ*mol Ni^2+^/1 kg b.w.	24 *μ*mol Zn^2+^/1 kg b.w.	24 *μ*mol Zn^2+^/1 kg b.w. and 19 *μ*mol Ni^2+^/1 kg b.w.

## Data Availability

The data supporting the findings of our study are available within the article.
